# Dynamic Fascial Closure With Vacuum-Assisted Wound Closure and Mesh-Mediated Fascial Traction (VAWCM) Treatment of the Open Abdomen—An Updated Systematic Review

**DOI:** 10.3389/fsurg.2020.577104

**Published:** 2020-11-05

**Authors:** Patrik Petersson, Ulf Petersson

**Affiliations:** ^1^Department of Clinical Sciences, Malmö, Faculty of Medicine, Lund University, Lund, Sweden; ^2^Department of Surgery, Skåne University Hospital, Malmö, Sweden

**Keywords:** open abdomen, negative pressure wound therapy (NPWT), vacuum assisted wound closure and mesh-mediated fascial traction, VAWCM, dynamic closure technique, temporary abdominal closure (TAC)

## Abstract

**Introduction:** Several different temporary abdominal closure techniques are described in the context of open abdomen treatment. Techniques based on dynamic fascial closure combined with negative pressure therapy have gained popularity and seem to result in the highest fascial closure rates without increased complications and are highlighted in recent guidelines and recommendations. One dynamic closure technique is the vacuum-assisted wound closure and mesh-mediated fascial traction (VAWCM) technique, first described in 2007. The aim of this systematic review was to evaluate the VAWCM technique regarding a number of short- and long-term results.

**Materials and Methods:** A systematic literature search was performed in PubMed, EMBASE, and Cochrane Library databases for articles published between January 1, 2006 and May 8, 2020. The review was independently performed by the two authors according to the PRISMA statements for reporting systematic reviews and meta-analyses. Results were pooled for presentation of weighted means when applicable.

**Results:** A total of 220 articles were screened by title and abstract. Thirty-two articles were assessed for eligibility by full-text review and 15 articles finally remained for review. A total of 600 patients treated with VAWCM were included. The pooled weighted means were as follows: fascial closure, 83.5%; enteroatmospheric fistula, 5.6%; planned ventral hernia, 6.2%; in-hospital survival, 72%; and incisional hernia incidence, 40.5%. Long-term survival ranged between 22 and 72%. Quality of life (SF-36) was reported in two studies showing lower scores than the population mean especially in physical domains. Incisional hernia resulted in lower scores in one but not in the other study.

**Discussion:** The results of 600 VAWCM-treated patients from 15 studies were evaluated in this systematic review. Earlier findings with high fascial closure rates, low enteroatmospheric fistula, and planned ventral hernia rates as well as high incisional hernia incidences were underlined. Permanent mesh for efficient fascial traction and reinforcement at fascial closure seem to be the next step in evolving an optimal temporary closure technique in open abdomen treatment.

## Introduction

Emergency conditions sometimes force surgeons to leave an abdominal incision unclosed and thereby initiating a period of open abdomen (OA) therapy. Meanwhile, a temporary abdominal closure (TAC) technique is used to protect the abdominal contents and to facilitate closure whenever intraabdominal and patient's overall condition is suitable. Causes for OA treatment can roughly be classified into four categories: (1) visceral edema and/or intraabdominal/retroperitoneal swelling with reduced intraabdominal space, making it mechanically impossible to close the abdomen; (2) intraabdominal deep infection/peritonitis needing active drainage; (3) damage control and/or planned second look operation; and (4) indication for decompression in case of abdominal hypertension or compartment syndrome (1). Due to the critical conditions in these patients, it is important that the utilized TAC technique minimizes the risk of complications related to the OA, since prolonged periods of treatment are associated with increased morbidity and mortality. Older static TAC techniques, e.g., Bogota bag or placement of a temporary mesh, did not facilitate closure and frequently resulted in large planned ventral hernias and concomitant morbidity.

With the introduction of negative pressure wound therapy (NPWT), the OA treatment techniques started to evolve. The novel vacuum-assisted wound closure and mesh-mediated facial traction (VAWCM) technique, combining negative pressure wound therapy and fascial traction, was described from our department in 2007 (2). The VAWCM technique was evaluated in a prospective multi-center cohort study presenting a fascial closure rate per protocol of 89%. However, long-term follow up showed a 54% incisional hernia (IH) incident in patients surviving 5 years, with a need for surgical repair in one third (3).

After the introduction of the VAWCM technique, several authors have adopted the technique and published their results. In a review article in 2017 (4), 11 studies evaluating the VAWCM technique was included with high fascial closure rates reported in most populations, while long-term IH development was only reported in 3. In these populations, high IH incidence after VAWCM was evident.

The European Hernia Society (EHS) published guidelines for OA treatment in 2018 (5), recommending the use of dynamic closure techniques. A recent review (6) on articles including short-term outcome of dynamic closure techniques published during the last 3 years updated the search done in the EHS guidelines and reported similar results for the different included dynamic closure techniques. In that review, the VAWCM technique dominated among the dynamic closure techniques. Furthermore, the World Society of Emergency Surgery (WSES) together with the Abdominal Compartment Society (WSACS) also recommend a dynamic closure technique with VAWCM to be used for OA treatment (7). The review and guideline articles share the conclusion that evidence in OA treatment is weak (5–7). A probable explanation is the low incidence of OA treatment per center together with the vast heterogeneity among OA patients and thereby great difficulties in performing randomized trials.

When fascial closure can be achieved in a high number of patients, the importance of evaluation of long-term results becomes evident. The purpose of this systematic review, performed in accordance with the PRISMA recommendations, is to update the present evidence for OA treatment with the VAWCM technique regarding short- and long-term results, with special attention to long-term outcome.

## Materials and Methods

Electronic database searches from January 1, 2006 to May 8, 2020 were conducted in MEDLINE (PubMed), EMBASE, and Cochrane Library Online with the purpose to identify all publications in English on OA treatment with the VAWCM technique. For detailed search terms, see Supplementary Material.

The review was performed according to the PRISMA (Preferred Reporting Items for Systematic reviews and Meta-Analyses) guidelines (8). The articles identified from the searches were initially screened for removal of duplicates and thereafter for inclusion on titles and abstracts. Full-text articles were assessed for eligibility whereafter the reference lists of these papers were scrutinized for additional eligible articles. Furthermore, reference lists from identified guideline articles on the matter were scrutinized. All articles, regardless of evidence level, were considered eligible for inclusion. Case reports with <5 patients, reviews, and guidelines were excluded. Furthermore, articles where major modifications of the VAWCM technique were utilized were excluded. The selection process was done by the two authors independently, and articles were included in mutual agreement.

Patient characteristics for the study populations (age, number of patients, and pathogenesis) were noted. Outcome variables of interest were fascial closure rate, time to fascial closure, number of dressing changes, enteroatmospheric fistula (EAF), and planned ventral hernia incidence, in-hospital survival and mortality, follow-up time, IH development and repair, long-time survival rate, and quality of life (QoL). Absence of data on some of the abovementioned outcome variables was not cause for exclusion. In some studies, another TAC technique besides VAWCM was used for some of the patients. From such articles, the results for VAWCM-treated patients were extracted and included.

### The Vacuum-Assisted Wound Closure and Mesh-Mediated Facial Traction (VAWCM) Technique

The VAWCM technique was described in detail by Petersson et al. (2). In summary, if an OA cannot be closed at the first dressing change, the mesh is applied and mesh-mediated fascial traction is started. A heavyweight polypropylene mesh is divided into two halves and sutured with a 2–0 running polypropylene suture with narrow bites to the fascial edges in an in-lay position on each side, whereafter the intraabdominal visceral protection layer of the NPWT system is applied. It is crucial that the visceral protection layer is tucked out as far laterally as possible to prevent adhesion formation between the intraabdominal content and the abdominal wall. The two mesh halves are thereafter pulled together under tension and sutured in the midline. The mesh-mediated tension on the abdominal wall prevents retraction of the lateral muscles, facilitating closure. The subcutaneous polyurethane foam is then placed between the abdominal wall edges, whereafter occlusive self-adhesive polyethylene films are applied to seal the wound. The tubing set is then applied, and the therapy unit of the NPWT system is set to −125 to −150 mmHg with continuous pressure.

Every 48–72 h, the abdominal dressing is changed. The tubing set, occlusive self-adhesive polyethylene films, and subcutaneous polyurethane foams are removed, and the mesh halves are opened in the midline. The intraabdominal visceral protection layer is removed. When the abdominal cavity has been carefully inspected and loose adhesions are divided, a new intraabdominal visceral protection layer is placed, and the mesh halves are re-sutured under tension in the midline. It is important to try to reduce the diastasis at each dressing change.

When the fascial edges can be aligned in the midline, the mesh is removed by cutting the running suture holding the mesh in the in-lay position and the incision is then closed by a running absorbable suture, carefully following the principles of a suture-to-wound ratio of 4:1.

### Statistics

Data from the different studies were pooled for description of weighted averages when applicable.

## Results

**Figure 1:**
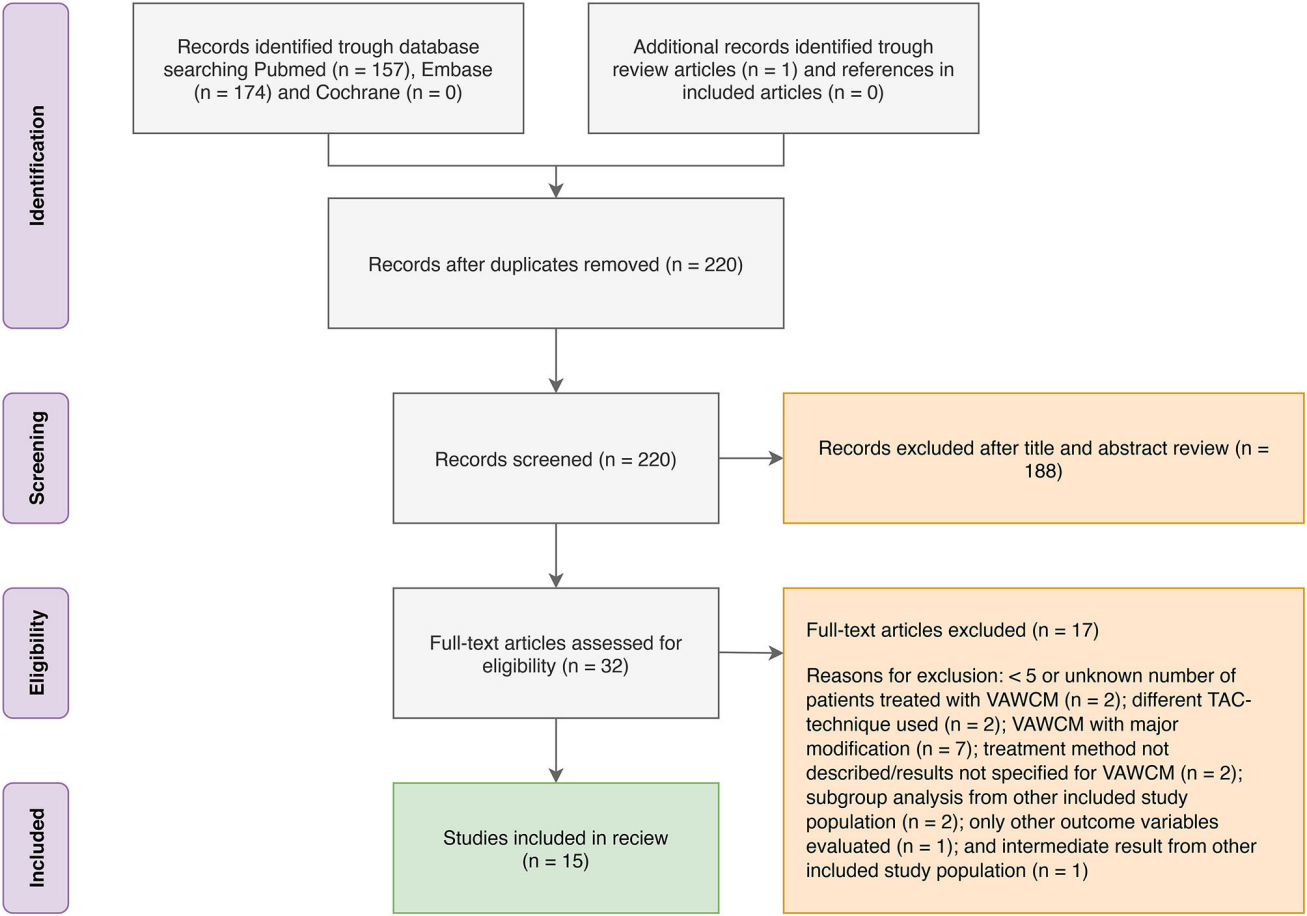
Flowchart for patient selection according to the PRISMA guidelines.

The MEDLINE (PubMed) search yielded 157 articles, the EMBASE search yielded 174 articles, and the Cochrane Library search yielded 0 articles. The identified articles were screened for duplicates, and the remaining 220 articles were screened by title and abstract. Of those, 32 articles were assessed for eligibility by full-text review. After exclusion, 15 articles were included in the review. Some of the articles only included parts of the outcome variables of interest for this review. Reasons for exclusion of full-text reviewed articles were as follows: <5 or unknown number of patients treated with VAWCM (*n* = 2); a different TAC technique was used (*n* = 2); VAWCM with major modification (*n* = 7); treatment method not described/results not specified for VAWCM (*n* = 2); subgroup analysis from other included study population (*n* = 2); only other outcome variables evaluated (*n* = 1); and intermediate result from other included study population (*n* = 1). For details, see the PRISMA flow diagram (Figure 1).

### Study and Patient Characteristics

Of the 15 articles included, 6 were prospective (3, 9–13) and 9 were retrospective (2, 14–21). No study was randomized, and most studies were observational with only one treatment group. Four reports (15, 18, 19, 21) included other techniques beside VAWCM, from which the VAWCM results were extracted. The study populations ranged between 7 and 111 with a mean of 40, and 600 patients were totally included (2, 3, 9–21). Thirteen articles included surgical patients, some in combination with vascular and/or trauma patients, one included only vascular patients, and the last article only included patients with peritonitis. For details on the included articles and patient characteristics, see Table 1.

**Table 1:** Included articles and patient characteristics.

**References**	**Article title**	**Author (year)**	**Study design**	**Inclusion period**	**VAWCM patients (*n*)**	**Type of patients**	**Age, years (median)**	**Long-term follow-up**
(2)	Vacuum-assisted wound closure and mesh-mediated fascial traction—a novel technique for late closure of the open abdomen	Petersson et al. (2007)	Retrospective	2005–2006	7	Surgical, vascular, trauma	65	No
(9)	Early results after treatment of open abdomen after aortic surgery with mesh traction and vacuum-assisted wound closure	Seternes et al. (2010)	Prospective	2006–2009	9	Vascular	70	Yes
(10)	Multicenter prospective study of fascial closure rate after open abdomen with vacuum and mesh-mediated fascial traction	Acosta et al. (2011)	Prospective	2006–2009	111	Surgical, vascular, trauma	68	No
(14)	Promising results after vacuum-assisted wound closure and mesh-mediated fascial traction	Kleif et al. (2012)	Retrospective	2009–2011	16	Surgical, non-trauma	66	No
(15)	Vacuum and mesh-mediated fascial traction for primary closure of the open abdomen in critically ill surgical patients	Rasilainen et al. (2012)	Retrospective	2008–2010	50	Surgical (ACS 47%)	60	No
(16)	Vacuum with mesh is a feasible temporary closure device after fascial dehiscence	Bjørsum-Meyer et al. (2013)	Retrospective	2008–2012	18	Surgical	64	Yes
(13)	Management of the open abdomen using vacuum-assisted wound closure and mesh-mediated fascial traction	Willms et al. (2015)	Prospective	2006–2012	53	Surgical, trauma	53 (mean)	No
(3)	Quality of life and hernia development 5 years after open abdomen treatment with vacuum-assisted wound closure and mesh-mediated fascial traction	Petersson et al. (2016)	Prospective	2006–2009	50	Surgical, vascular, trauma	70	Yes
(17)	Retrospective analysis of a VACM (vacuum-assisted closure and mesh-mediated fascial traction) treatment manual for temporary abdominal wall closure—results of 58 consecutive patients	Beltzer et al. (2016)	Retrospective	2007–2008	31	Surgical	67	No
(11)	Abdominal wall integrity after open abdomen: long-term results of vacuum-assisted wound closure and mesh-mediated fascial traction (VAWCM)	Willms et al. (2016)	Prospective	2006–2013	34	Surgical, trauma	56 (mean)	Yes
(18)	Greater success of primary fascial closure of the open abdomen: A retrospective study analyzing applied surgical techniques, success of fascial closure, and variables affecting the results	Kääriäinen et al. (2017)	Retrospective	2009–2013	30	Surgical, Vascular	–	No
(19)	Open abdomen treated with negative pressure wound therapy: Indications, management and survival	Seternes et al. (2017)	Retrospective	2006–2014	92	Vascular, surgical, trauma (ACS 44%)	–	No
(20)	Open abdomen with vacuum-assisted wound closure and mesh-mediated fascial traction in patients with complicated diffuse secondary peritonitis: A single-center 8-year experience	Tolonen et al. (2017)	Retrospective	2008–2016	41	Peritonitis	59	No
(12)	Intensive care and health outcomes of open abdominal treatment: long-term results of vacuum-assisted wound closure and mesh-mediated fascial traction (VAWCM)	Willms et al. (2017)	Prospective	2006–2013	27	Surgical, trauma	56 (mean)	Yes
(21)	Blurring the boundary between open abdomen treatment and ventral hernia repair	Käser et al. (2019)	Retrospective	2013–2015	31	Surgical, septic peritonitis	58	Yes

### Short-Term Results

Twelve of fifteen articles reported on short-term outcomes (see Tables 2, **4**). The fascial closure rate per protocol varied between 50 and 100% (2, 9, 10, 13–21) with a pooled weighted average rate, for 11 studies, of 83.5%. Time to fascial closure varied between 7 and 32 days, and the number of dressing changes between 2 and 10. EAF development was seen in 0–12% (2, 9, 10, 13–17, 20) with a pooled weighted average of 5.6% and planned ventral hernia incidence varied between 0 and 50% (2, 9, 10, 13–16, 18, 20, 21) with a pooled weighted average of 6.2%. The pooled in-hospital survival was 72% with range between 55 and 87% (2, 9, 10, 13–17, 20).

**Table 2:** Short-term outcome.

**References**	**Author (year)**	**Patients alive at OA closure**	FC per protocol, %^*^	**Time to closure, days (median)**	**Dressing changes, *n* (median)**	EAF (%)^*^	**Closure with mesh bridging (%)^†^**	**Closure with adjunct CS (%)******	**Small fascial defect at closure (%)^**±**^**	Planned ventral hernia^*^	In-hospital survival (%)^*^
(2)	Petersson et al. (2007)	7	100	32	10	0	0	0	0	0	86
(9)	Seternes et al. (2010)	8	100	10.5	3	0	0	0	0	0	66
(10)	Acosta et al. (2011)	95	89.5	14	4	6.3	8.4	0	2.1	0	70
(14)	Kleif et al. (2012)	14	50	10	4	0	0	0	0	50	87
(15)	Rasilainen et al. (2012)	42	92.9	9	3.5	12	0	0	0	7.1	62
(16)	Bjørsum-Meyer et al. (2013)	15	80	21	3	0	13.3	0	0	6.7	83
(13)	Willms et al. (2015)	47	89.4	10	6.2 (mean)	1.8	0	0	0	10.6	87
(17)	Beltzer et al. (2016)	31	61	–	–	6.5	–	–	–	–	55
(18)	Kääriäinen et al. (2017)	30	83.3	20.6 (mean)	–	–	10	0	0	6.7	–
(19)	Seternes et al. (2017)	–	84	–	–	–	–	–	–	–	–
(20)	Tolonen et al. (2017)	36	83.3	7	2	7.3	0	8.3	2.8	5.6	71
(21)	Käser et al. (2019)	31	58	–	5	–	42	0	0	0	–

FC, Facia closure; EAF, enteroatmospheric fistula; CS, component separation.

* Outcome included in the pooled data presented in Table 4.

† Closure with mesh bridging when fascial closure was not possible. ^**‡**^Facial closure without mesh was achieved after component separation.

± *Fascial closure achieved in major part of the incision with smaller fascial defect remaining*.

### Long-Term Results

Six of fifteen articles reported on long-term outcomes (see Tables 3, 4). The follow-up time was 17–63 months (3, 9, 11, 12, 16, 21). IH rate was 21–54% (3, 9, 11, 16, 21), and the pooled weighted average was 40.5%. IH repair, in the two studies reporting on this, was 33 and 42%, respectively (3, 11). The survival rate at follow-up was reported in four studies and ranged between 22 and 72% (3, 11, 16, 21). Two articles presented data on QoL using the SF-36 questionnaire. In one of the studies (3), both component scores and all subscales except bodily pain were lower than the population mean and correlated with major comorbidity and the presence of a stoma. In that study, no differences in SF-36 scores were found between patients with and without an IH. Neither did abdominal wall discomfort differ in relation to IH, when evaluated with the Ventral Hernia Pain Questionnaire. In the other study (12), lower scores for role physical, physical function, and physical component score were reported and correlated with the complex intensive care score being a surrogate marker of severity of global illness. Patients with an IH scored lower than the total study population as well as the population mean in the same domains.

**Table 3:** Long-term results.

**References**	**Author (year)**	**Follow-up time, months (median)**	**Patients eligible for follow-up (*n*)**	IH after FC (%)^*^	IH repair (% of IH)^*^	**Long-term survival (%)**	**QoL comments**
(9)	Seternes et al. (2010)	17	6	38	–	–	–
(16)	Bjørsum-Meyer et al. (2013)	21	14	21	–	72.2	–
(3)	Petersson et al. (2016)	63	50	54	33	49.5	SF-36: Generally lower scores except BP. No difference between IH and non-IH.
(11)	Willms et al. (2016)	46	34	35	42	73	–
(12)	Willms et al. (2017)	46	–	–	–	–	SF-36: Lower physical domains. Hernia patients had lower PF, GH, and PCS than others.
(21)	Käser et al. (2019)	24	9	22	–	22	–

IH, Incisional hernia; QoL, Quality of Life.

* *Outcome included in the pooled data presented in Table 4*.

**Table 4:** Pooled data.

**Outcome variable**	Pooled result^*^ (%)
Fascia closure rate per protocol	83.5
Enteroatmospheric fistula	5.6
Planned ventral hernia	6.2
In-hospital survival	72.0
Incisional hernia after fascia closure	40.5
Incisional hernia repair	35.8

* *See Tables 2, 3 for included article*.

## Discussion

This is an update of the review on VAWCM-treated patients published in 2017 (4). In this review, five additional studies meeting our inclusion criteria have been included (12, 14, 17, 20, 21), adding data on both short- and long-term outcomes. Fifteen articles were included in this review displaying great heterogeneity among patients with different pathogeneses, comorbidities, and causes for OA therapy.

Optimization in the management of OA patients, whether treated with VAWCM or other TAC techniques, is of fundamental importance for the results and must be emphasized. The desire to close the OA as quickly as possible, to prevent complications induced by the OA as such, must be balanced against organ dysfunction needing further decompression, the possibilities of accomplishing negative fluid balance for reducing visceral edema, the need of further drainage, or delayed reconstructive measures. While taking this into consideration, the surgical performance needs to be optimized. For VAWCM, this implies starting the traction early during OA treatment and performing every dressing change and mesh tightening procedure in time with the intention and skill to reduce the fascial diastasis successively, i.e., every 2–3 days, daytime by a surgeon familiar with the technique and for a long enough period of time to achieve fascial medialization and closure. By own experience, we know that it can be hard to comply with these prerequisites for optimal utilization of the VAWCM technique, which, however, must be strived for in order to improve outcome. Besides the almost unanimous compliance with dressing change intervals, it is not possible to evaluate the other important factors in the included studies.

The review revealed per-protocol fascial closure rates between 50 and 100%. Failure of fascial closure necessitates an alternative measure when terminating the OA. The alternatives utilized in the included studies have been leaving the patient with a large planned ventral hernia, closure with mesh bridging, or fascial closure with component separation. The incidence of planned ventral hernias varied between 0 and 50% (2, 9, 10, 13–16, 18, 20, 21) with a pooled weighted average of 6.2%. The use of mesh bridging (10, 16, 18, 21) or component separation (20) lowered the planned ventral hernia rate but was not part of the basic idea of the VAWCM technique and must be considered a failure in evaluation of the technique. This vast variation in closure and planned ventral hernia rates might depend on patient and pathogenic heterogeneity, but the ability to utilize and perform the technique in an optimal way in each patient is likely to contribute to the differences to an even larger extent. Nevertheless, the high weighted average closure rate of 83.5% must be considered a good result, implying that the VAWCM technique is reproducible and relatively simple.

A feared complication during OA therapy is the development of an EAF, which has been shown to be a significant predictor of failure of fascial closure and possibly also of mortality in OA patients (10, 22) even if an interim analysis from the International Registry of Open Abdomen did not find any EAF impact on mortality (23). A word of caution was raised when NPWT for OA treatment was popularized (24, 25). A damaging effect of the negative pressure, supposed to propagate to the bowel surface, was proposed to be the pathogenic mechanism. With use of a visceral protective layer, it appears as the negative pressure propagation to the bowel surface is significantly reduced, independently of preset negative pressure, according to the results of an experimental porcine study (26). The concern for NPWT-induced EAF development has later been toned down as a result of increased experience with the technique and succeeding publications stating that the use of NPWT in OA patients, on the contrary, seems to reduce the incidence of EAF compared to non-NPWT-treated patients, especially when combining NPWT with fascial traction (27). In this review, the weighted average for EAF formation was 5.6% (range 0–12%), which is in the lower range of earlier published results for OA treatment, regardless of treatment technique (23, 27). Multiple studies have, as shown in this review, evaluated the VAWCM technique without finding proof of problematic EAF formation rates.

The weighted average for in-hospital survival for patients treated with VAWCM in this review was 72%, i.e., 28% in-hospital mortality, which is in line with other reports. For comparison, a systematic review (28) reported in-hospital mortality rates, from 12 studies including 2733 patients, between 14 and 59% with a weighted average mortality rate of 31.3%.

The short-term results in this updated review strengthen the previously found high fascial closure rates, low planned ventral hernia and EAF rates.

Long-term results were reported in six articles with median follow-up of 17–63 months. IH rates were reported in five studies ranging from 21 to 54% at the latest follow-up occasion (3, 9, 11, 16, 21), resulting in a weighted average of 40.5%. Clinical examination was the basis for IH diagnosis in three of the studies. In two of the three prospective studies, the protocol included a CT scan (3) or an ultrasound (11) contributing to higher sensitivity in IH diagnosis (3). These two studies had a reasonable number of patients eligible for follow-up and reported 54 and 35% IH, respectively (3, 11). The high IH rates and the resulting 30–40% IH repair rate from the former review on the technique was thereby underlined. IH rates in the same range have been reported after use of other fascial traction techniques not including any fascial reinforcement at definitive closure (29, 30).

Long-term survival varied largely, from 22 to 73%, in the four studies reporting on this (3, 11, 16, 21). This most certainly accounts for differences in age, comorbidities, and causes for OA treatment, and a major loss to follow-up must be anticipated whenever OA long-term results are to be evaluated.

Only two articles reported on QoL, and both showed that patients treated with OA had lower SF-36 scores than the population mean (3, 12), but the presence of an IH influenced the scores negatively only in one of the studies. In one of the studies (3), the scores were overall lower than the Swedish population mean and correlated with major comorbidity and the presence of a stoma but not with the presence of an IH. Furthermore, no differences in abdominal wall discomfort were found between patients with and without an IH when evaluated with the Ventral Hernia Pain Questionnaire. In the other study (12), lower scores than the German population mean for physical domains were reported and correlated with the complex intensive care score being a surrogate marker of severity of global illness. Patients with an IH scored lower than the total study population as well as population mean in the same domains. In view of the sparse information on QoL after OA treatment found in the literature, it is of importance to include QoL evaluation in upcoming study protocols.

Articles reporting on long-term results was six, which is twice the number compared to the earlier review (4). A high IH rate was underlined, but data on QoL were only added from one study indicating a lower QoL after OA treatment than in the population mean.

Reporting weighted averages or pooled outcomes provides a more accurate view of the combined results from many studies with a wide range of included patients, but this review also has several weaknesses attached. No article on the VAWCM technique was randomized, and more than half of the articles were retrospective with low to very low evidence level. Heterogeneity in pathogenesis, severity of illness, and patient characteristics together with a relatively infrequent use of OA at a single institution are reflected in the quality of many of the included studies and also inflict problems in conducting good RCTs and thereby improve the level of evidence in this research area. Four reports (15, 18, 19, 21) included other techniques beside VAWCM, and results for VAWCM had to be extracted, which may inflict a risk of misinterpretation of data. Intention-to-treat analyses were not possible due to missing data, and therefore, results per protocol on patients surviving until fascial closure was attempted were the only option for many of the outcome variables. There is also a minor risk that a small number of patients may be reported in more than one article, but where the suspicion was obvious, only one of the reports were included.

### Conclusion

Dynamic fascial closure combined with NPWT, as in the VAWCM technique, seems to provide the best OA treatment results according to today's knowledge, albeit mostly based on weak evidence (5–7). The VAWCM technique is not the only technique based on this combination but mesh-mediated fascial traction plus NPWT is today best evaluated with high fascial closure rates, low EAF and planned ventral hernia rates, but high IH rates. The future challenges for mesh-mediated fascial traction plus NPWT-based techniques are short term to further increase fascial closure and long term to reduce IH rates. Permanent mesh for traction and reinforcement at fascial closure may solve both of these problems but need to be prospectively evaluated (1, 31, 32).

## Data Availability Statement

The original contributions generated for the study are included in the article/Supplementary Material, further inquiries can be directed to the corresponding author/s.

## Author Contributions

PP and UP: contribution to the conception of the work, the acquisition, analysis, and interpretation of data, drafting the work, final approval of the version to be published, and agreement to be accountable for all aspects of the work in ensuring that questions related to the accuracy or integrity of any part of the work are appropriately investigated and resolved. All authors contributed to the article and approved the submitted version.

## Conflict of Interest

The authors declare that the research was conducted in the absence of any commercial or financial relationships that could be construed as a potential conflict of interest.
