# Supplementary material for: Dynamic Fascial Closure With Vacuum-Assisted Wound Closure and Mesh-Mediated Fascial Traction (VAWCM) Treatment of the Open Abdomen—An Updated Systematic Review
Source: Front Surg. 2020 Nov 5;7:577104. doi: 10.3389/fsurg.2020.577104 (PMC7674165; doi:10.3389/fsurg.2020.577104)
Supplement: Supplementary file 1 [file Data_Sheet_1.DOCX]

Supplementary Material

# Search-terms

The following search-term builds were used:

MEDLINE (PubMed);

(((VAWCM) OR (vacuum-assisted wound closure and mesh-mediated fascial traction)) OR (((open abdomen) OR (temporary abdominal closure) OR (TAC) OR (wound dehiscence) OR (fascia* dehiscence) OR (burst abdomen) OR (decompres* laparotomy) OR (laparostomy) OR (laparotomy) OR (delayed abdominal closure) OR (abdominal compartment syndrome) OR (ACS)) AND ((vacuum assisted wound closure) OR (vacuum-assisted wound closure) OR (negative wound therapy) OR (negative pressure therapy) OR (negative pressure wound therapy) OR (NPWT) OR (vacuum*)) AND ((mesh) OR (mesh-mediated fascial traction) OR (mesh mediated fascial traction)))) AND ("2006/01/01"[Date - Publication] : "2020/05/08"[Date - Publication]) AND (english[Language])

EMBASE;

(((VAWCM) OR (vacuum-assisted wound closure and mesh-mediated fascial traction)) OR (((open abdomen) OR (temporary abdominal closure) OR (TAC) OR (wound dehiscence) OR (fascia* dehiscence) OR (burst abdomen) OR (decompres* laparotomy) OR (laparostomy) OR (laparotomy) OR (delayed abdominal closure) OR (abdominal compartment syndrome) OR (ACS)) AND ((vacuum assisted wound closure) OR (vacuum-assisted wound closure) OR (negative wound therapy) OR (negative pressure therapy) OR (negative pressure wound therapy) OR (NPWT) OR (vacuum*)) AND ((mesh) OR (mesh-mediated fascial traction) OR (mesh mediated fascial traction)))) with filter for English language and dates 2006-01-01 to 2020-05-08

Cochrane Library Online;

"VAWCM" OR "vacuum-assisted wound closure and mesh-mediated fascial traction" OR "open abdomen" OR "temporary abdominal closure" OR "TAC" OR "wound dehiscence" OR "fascia dehiscence" OR "fascial dehiscence" OR "burst abdomen" OR "decompress laparotomy" OR "laparostomy" OR "laparotomy" OR "delayed abdominal closure" OR "abdominal compartment syndrome" OR "ACS" in Title Abstract Keyword AND "vacuum assisted wound closure" OR "vacuum-assisted wound closure" OR "negative wound therapy" OR "negative pressure therapy" OR "negative pressure wound therapy" OR "NPWT" OR "vacuum" in Title Abstract Keyword AND "mesh" OR "mesh-mediated fascial traction" OR "mesh mediated fascial traction" in Title Abstract Keyword - with Cochrane Library publication date Between Jan 2006 and May 2020 (Word variations have been searched)
